# Serum IL-10 as a marker of severe dengue infection

**DOI:** 10.1186/1471-2334-13-341

**Published:** 2013-07-24

**Authors:** Gathsaurie Neelika Malavige, Laksiri Gomes, Lukmall Alles, Thashi Chang, Maryam Salimi, Sachie Fernando, Kushan DL Nanayakkara, SD Jayaratne, Graham S Ogg

**Affiliations:** 1 Department of Microbiology, University of Sri Jayawardanapura, Nugegoda, Sri Lanka; 2 Department of Medicine, Faculty of Medical Sciences, University of Sri Jayawardanapura, Nugegoda, Sri Lanka; 3 Department of Clinical Medicine, Faculty of Medicine, University of Colombo, Colombo, Sri Lanka; 4 MRC Human Immunology Unit, Weatherall Institute of Molecular Medicine, Oxford NIHR Biomedical Research Centre and University of Oxford, OX3 9DS, Oxford, UK

## Abstract

**Background:**

Several studies have shown that serum IL-10, IFNγ and MIF are elevated in patients in severe dengue (SD) and could be used as potential biomarkers. We proceeded to determine if these cytokines could be used as biomarkers in a large cohort of adult dengue patients with varying severity of dengue infection.

**Methods:**

Serum IL-10 levels were determined in 259 of whom 40 had severe dengue infection. Serum IFNγ and IFNα levels were done in 78 and MIF levels were done in 65 patients with acute dengue infection. Clinical features and laboratory investigations were undertaken during the febrile and critical phase.

**Results:**

We found that serum IL-10 levels were significantly higher (p = 0.001) in patients with SD, when compared to those with non SD. Serum IL-10 levels significantly and inversely correlated with white cell counts (R = −0.23, p = 0.0002) and lymphocyte counts (R = −0.29, p < 0.0001) but significantly and positively correlated with aspartate tranaminase levels (R = 0.16, p = 0.01) and alanine transaminase levels (R = 0.22, p = 0.007). However, IL-10 levels did not have a good predictive value in discriminating those who were likely to develop SD (AUC = 0.66). Serum IFNγ levels were also significantly higher (p = 0.04) in patients with SD when compared to non SD. There was no difference (p = 0.34) in serum IFNα levels and serum MIF levels (p = 0.15) in patients with SD and non SD.

**Conclusion:**

Although serum IL-10 was significantly elevated in patients with SD it had a poor discriminatory value in identifying those with SD and non SD and therefore, is unsuitable to be used as a robust biomarker in this cohort.

## Background

Dengue viral infections have become one of the most important mosquito borne viral infections in the world with a 30-fold increase incidence. It has caused significant outbreaks in five of six World Health Organization (WHO) regions. It is estimated that 2.1 million cases of dengue haemorrhagic fever (DHF)/dengue shock syndrome (DSS) occur every year [1]. Sri Lanka has had several annual dengue epidemics for the past 21 years, and the incidence and severity of these epidemics is increasing [2].

Although most individuals infected with the dengue virus experience asymptomatic or mild febrile illness, some develop dengue haemorrhagic fever (DHF) or shock (DSS) which can be fatal. Case fatalities due to dengue infection are around 1-5% depending on the country and epidemic [3]. Early detection of shock and other complications and supportive therapy has shown to reduce the morbidity and mortality [3-5]. Dengue infections predominantly affect developing countries which have scarce resources to handle large numbers of patients who are admitted to wards during periods of epidemics. Therefore, early identification of patients who are likely to develop severe disease and complications would enable clinicians to act quickly in order to minimize severe disease.

Clinical features in dengue infection occur abruptly and are often described according to three phases- the febrile phase, critical phase and the convalescent phase. Severe clinical disease manifestations such as fluid leakage, bleeding and shock are seen during the critical phase which begins around day 4–7 of the illness and usually lasts 48–72 hours [3]. However, not all patients who are in the febrile phase proceed to the critical phase and some proceed directly to the convalescent phase. An ideal biomarker should be able to identify which patients are likely to develop severe clinical disease manifestations, before they develop bleeding, fluid leakage or shock.

Several groups have tried to identify cytokine and other laboratory profiles in patients with varying severity of dengue infection, in order to determine which of these could be used as predictors for development of severe clinical disease. In these studies, IL-1ß, IFN-γ, IL-4, IL-6, IL-13, IL-7, GM-CSF (Granulocyte-macrophage colony-stimulating factor), MIF (macrophage migration inhibitory factor), IL-10 along with several other cytokines have been found to be elevated in patients with DHF when compared to dengue fever (DF) [6-10]. Some have shown that MIF, IL-10, IL-6, MIP-1ß and IFNγ could be used as potential predictors of severe dengue [6,9]. As dengue infections are very dynamic, more recent studies have investigated serial changes in cytokine patterns in patients with acute dengue infection. A recent very carefully conducted study in 44 adult patients with dengue fever DF and 18 patients with DHF has showed that cytokines such as IFNγ, IL-17, IL-4, IL-1β and vascular endothelial growth factor (VEGF) were significantly higher in patients with DF when compared to those with DHF in the febrile phase [10]. In this study where all patients had primary dengue, all cytokine levels were higher in patients with DF when compared to those with DHF. Similar studies conducted in a larger group of children with DF and DHF have shown that serum IL-10 levels rose at defervescence only in those who progressed to DSS [11]. Other longitudinal studies have also shown that serum IL-10 levels increased during defervescence in dengue patients with warning signs, but IL-10 levels fell in those with no warning signs [12].

As our previous studies have shown that IL-10 was associated with T cell apoptosis [13] and many other studies have shown that serum IL-10, IFNγ and MIF levels were higher in patients with severe dengue we proceeded to investigate the use of these cytokines as potential biomarkers to predict those who are likely to develop severe disease in a large cohort of infected adult individuals.

## Methods

### Patients

259 adult patients with clinical features suggestive of dengue infection who were admitted to a general medical ward in two tertiary care hospitals in Colombo during the year 2011, were enrolled in the study following informed written consent. Blood samples were also obtained from 15 healthy volunteers to determine serum cytokine levels in the population. The study was approved by the Ethical Review Committee of the University of Sri Jayawardanapura. The initial blood sample was obtained during day 4 and 5 of illness (day 1 was considered as first day of fever). A second blood sample was obtained in 65/259 patients during the critical phase (2 days after obtaining the first blood sample) in those who gave consent. Clinical features such the presence of dengue warning signs, blood pressure, pulse rate and volume, general status of the patient, presence of any bleeding manifestations and presence of any possible fluid accumulation in the pleural cavity and abdomen were monitored several times a day from the time of admission to hospital, until they were discharged. Laboratory investigations such as the full blood counts were done several times a day in all patients until they were discharged from hospital. Serum electrolytes, coagulation profiles, chest radiography and ultrasound scans of the abdomen were only done in selected patients due to resource limitations. The patients were classified as having severe dengue according to the 2009 WHO guidelines [3]. Based on the 2009 WHO diagnostic criteria, shock was defined as lowering of pulse pressure to 20 mmHg or less or the presence of signs of poor capillary perfusion (cold extremities, poor capillary refill or a rapid pulse rate) [3]. Patients were classified as having severe dengue if they developed signs of shock, developed severe bleeding or had features of organ impairment such as liver transaminases exceeding 1000/IU.

### Serology

Acute dengue infection was confirmed by testing the serum samples with a commercial capture-IgM and IgG enzyme-linked immunosorbent assay (ELISA) (Panbio, Brisbane, Australia). The ELISA was performed and the results were interpreted according to the manufacturer’s instructions. This ELISA assay has been validated as both sensitive and specific for primary and secondary dengue virus infections [14,15].

### Quantitative cytokine assays

Quantitative cytokine assays were done in duplicate on serum samples obtained on 4–5 of illness. Serum IL-10 levels (Mabtech, Sweden) were done in all serum samples and were also done in all the serum samples obtained during the critical phase using IL-10 pre-coated ELISA plates according to the manufacturer’s instructions. Serum IFNγ levels (Mabtech, Sweden) and IFNα levels (Mabtech, Sweden) were only done in duplicate in 78 patients in the samples obtained on the date of admission and these assays were again performed using precoated IFNγ ELISA plates and precoated IFNα ELISA plates according to the manufacturer’s instructions. Serum MIF levels (Biolegend, USA) were done in duplicate in paired serum samples obtained on the date of admission and the critical phase (2 days after obtaining the first blood sample) using MIF pre-coated ELISA plates according to the manufacturer’s instructions.

### Statistical analysis

Statistical analysis was performed using Graph pad PRISM version 6. As the data were not normally distributed, differences in means were compared using the Mann–Whitney U test (two tailed). Degree of association between clinical parameters and serum cytokine levels were done using Spearmans correlation. Receiver-operator characteristic (ROC) curves showing the area under the curve (AUC) were generated to determine the discriminatory performance of serum IL-10 levels, platelet counts, lymphocyte counts and liver transaminase levels.

## Results

Of the 259 patients, 40 (15.4%) patients were classified as having severe dengue. Their mean age was 26.8 years (SD ± 11.9). 72 (27.8%) had abdominal pain, 100 (38.6%) had vomiting, 37 (14.3%) had pleural effusions, 7 (2.7%) had ascites and 17 (6.6%) developed bleeding manifestations. However, none of the patients developed severe bleeding. Based on the presence of dengue specific IgM antibodies in the absence of IgG antibodies, 50 (19.3%) patients were found to have primary dengue infection.

### Association of serum IL-10 with disease severity

**Figure 1:**
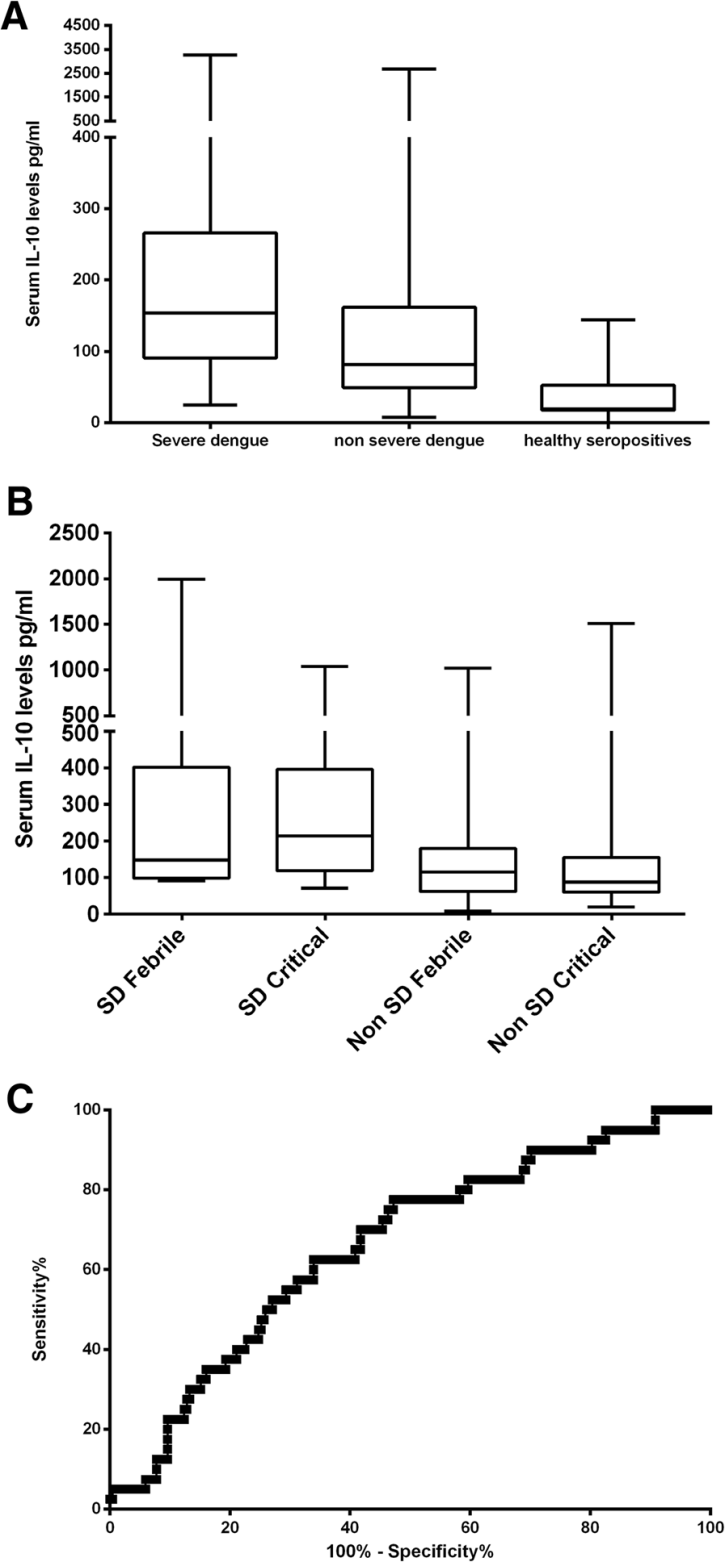
**Serum IL-10 levels and clinical diseases severity. A**: Serum IL-10 levels in patients with severe dengue (n = 40), non severe dengue (n = 219) and healthy controls (n = 11). **B**: Serum IL-10 levels in patients with severe dengue (SD) (n = 7) and non severe dengue (n = 57) in the febrile phase and the critical phase. **C**: ROC curves of serum IL-10 levels (n-259).

Patients with severe dengue had significantly lower white cell counts and platelet counts and significantly higher alanine transaminase levels and aspartate transaminase levels than those with non severe dengue (Table 1). Serum IL-10 levels were significantly higher (p = 0.001) in patients with severe dengue (mean 291.6 ± SD 573.6, median 154.3, range 24.98 to 3271 pg/ml), when compared to those with non severe dengue (mean 156.6 ± SD 258.2, median 81.9, range 7.8 to 2681 pg/ml) (Figure 1A). Serum IL-10 levels significantly and inversely correlated with white cell counts (Spearmans R = −0.23, p = 0.0002) and lymphocyte counts (Spearmans R = −0.29, p < 0.0001). Serum IL-10 levels significantly and positively correlated with the highest aspartate tranaminase (AST) levels (Spearmans R = 0.16, p = 0.01) and the highest alanine transaminase (ALT) levels (Spearmans R = 0.22, p = 0.007) but not with the lowest platelet count in the critical phase (Table 2).

**Table 1:** Laboratory parameters in patients with severe and non severe dengue

**Clinical characteristic**	**Severe dengue N = 40**	**Non severe dengue N = 219**	**P value**
	**Mean (±SD)**	**Mean (±SD)**	
Day of illness when admitted to hospital	4.8 (±1.2)	5.25 (±1.6)	0.17
Age (years)	23.3 (8.8)	27.4 (11.4)	0.03
Lowest white cell count (cells/mm^3^)	2761 (±1406)	3434 (±2103)	0.059
Lowest lymphocyte count (cells/mm^3^)	1060 (±579)	1507 (±1230)	0.06
Lowest platelet count (cells/mm^3^)	29,949 (±26,335)	47,229 (±33,691)	0.0004
Alanine transaminase levels	213.8 (±279.9)	87.03 (±95.05)	0.001
Aspartate transaminase levels	474.6 (±893.2)	169.9 (±154.4)	0.0001
Serum IL-10 levels pg/ml	291.6 (±573.8)	183.4 (±472.8)	0.001

**Table 2:** Correlation of laboratory parameters with serum IL-10 levels in the febrile phase

**Laboratory parameters**	**Spearmans R value**	**P value**
Lowest lymphocyte count	−0.23	0.0002
Lowest white cell count	−0.29	<0.0001
Lowest platelet count	0.03	0.55
Aspartate transaminase levels	0.16	0.02
Alanine transaminase levels	0.22	0.0007

Serum IL-10 levels in the febrile phase, very significantly and positively correlated with serum IFNγ levels (Spearmans R = 0.55, p < 0.0001) and IFNα levels (Spearmans R = 0.3, p = 0.04) in the febrile phase. Serum IL-10 and MIF levels were determined in 65 patients (7 of them with severe dengue) in both the febrile phase and the critical phase. Serum IL-10 levels rose in 17/65 of these patients, while serum IL-10 levels fell in the other 48 patients (Figure 1B). Of the 17 patients in which the serum IL-10 levels rose, 5 had severe dengue. There was no difference in other parameters such as platelet counts, white cell counts and liver transaminases in patients in whom the IL-10 levels rose in the second sample when compared to other patients. However, serum IL-10 levels in the critical phase were again significantly higher (p = 0.01), in patients with SD (mean 323.1, SD ± 331.9, median 214.2, range 71.82 to 1036 pg/ml) when compared to those with non SD (mean 147.3, SD ± 217.5, median 87.9, range 20.5 to 1508 pg/ml). Serum IL-10 levels in the febrile phase significantly and positively correlated with the paired IL-10 levels in the critical phase (Spearmans R = 0.67, p < 0.0001). Although the serum IL-10 levels in the febrile phase did not correlate with MIF levels in the febrile phase, they negatively correlated with serum MIF levels observed in the critical phase (Spearmans R = −0.47, p = 0.01) (Table 3).

**Table 3:** Correlation of serum IL-10 levels in the febrile phase with other serum cytokines

**Cytokine**	**Spearmans R value**	**P value**
Serum IL-10 levels in the critical phase	0.67	<0.0001
Serum MIF levels in the febrile phase	0.06	0.61
Serum MIF levels in the critical phase	−0.47	0.01
Serum IFNγ levels in the febrile phase	0.55	<0.0001
Serum IFNα levels in the febrile phase	0.29	0.04

### Association of other cytokines with disease severity

**Figure 2:**
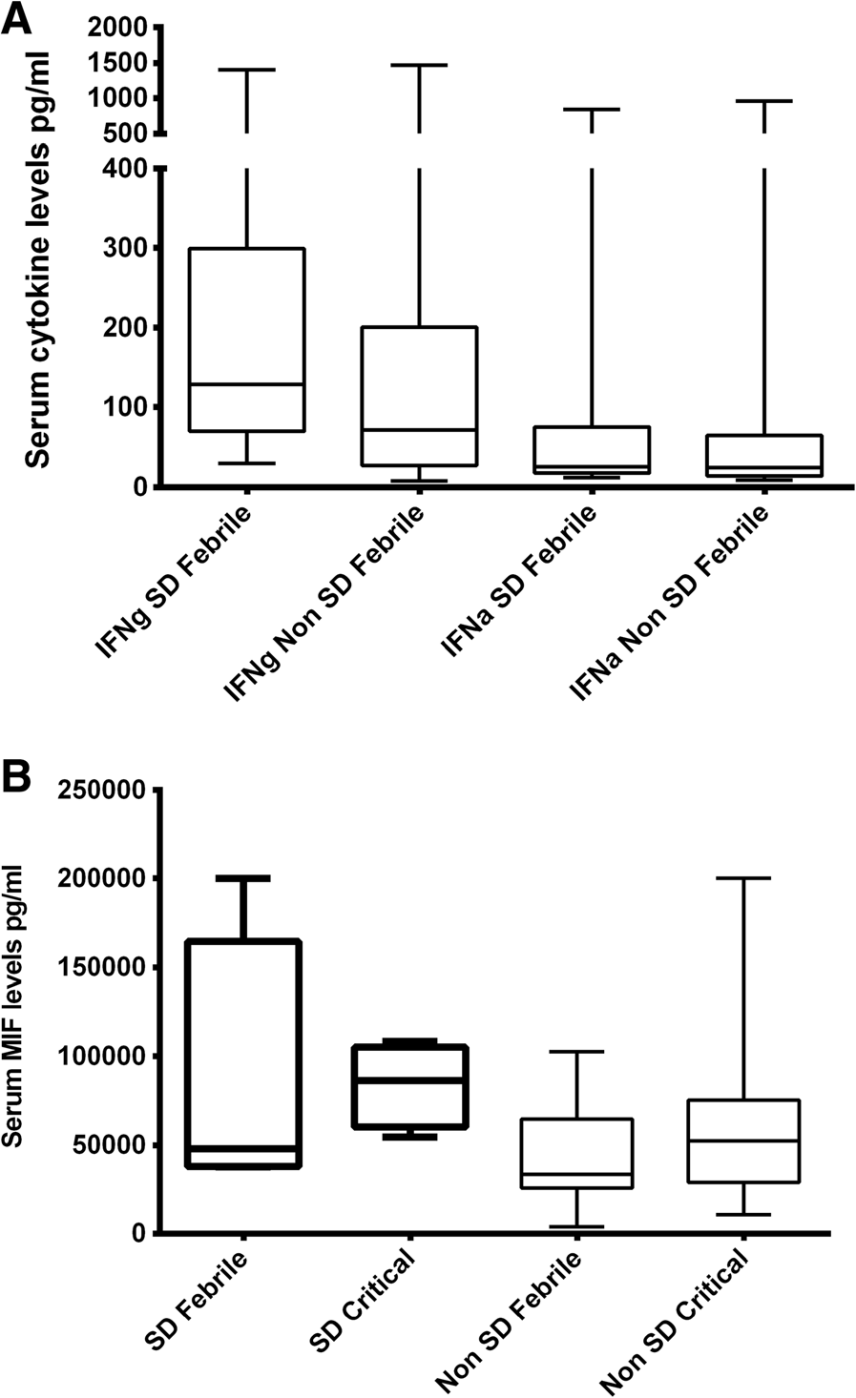
**Association of serum interferon and MIF levels with clinical disease severity. A**: Serum IFNγ in patients with severe dengue (n = 20) and non severe dengue (n = 58) and IFNα levels in patients with severe dengue (SD) (n = 20) and non SD dengue in the febrile phase (n = 58). **B**: Serum MIF levels in patients with severe dengue (SD) (n = 7) and non severe dengue (n = 57) in the febrile phase and the critical phase.

Serum IFNγ levels were also significantly higher (p = 0.04) in patients with severe dengue (mean 268.8, SD ± 346.2, median 128.4, range 29.8 to 1405 pg/ml) when compared to non severe dengue (mean 174, SD ± 265.6, median 72.2, range 8.1 to 1466 pg/ml). (Figure 2A). Serum IFNγ levels significantly and negatively correlated with white cell counts (Spearmans R = −0.37, p = 0.001) and lymphocyte counts (Spearmans R −0.44, p = 0.0001). However, no association was seen with liver transaminases or platelet counts. There was no difference (p = 0.34) in serum IFNα levels in patients with SD (mean 102, SD ± 201, median 26.14, range 12.37 to 838.2 pg/ml) and non SD (mean 83.33, SD ± 167.4, median 25, range 8.9 to 960.1 pg/ml) (Figure 2A). However, as seen with serum IL-10 and IFNγ levels a significant and negative correlation was observed with serum IFNα levels and white cell counts and lymphocyte counts.

Serum MIF levels were done in 65 paired serum samples. Serum MIF levels were higher in patients with severe dengue (mean 69,007, SD ± 58,692, median 48,044, range 12,685 to 200,000 pg/ml) when compared to those with non severe dengue (mean 39,783, SD ± 26,139, median 28,932, range 3908 to 102,589 pg/ml) although this was not statistically significant (p = 0.15) (Figure 2B).

### Serum cytokines in primary vs secondary dengue

**Figure 3:**
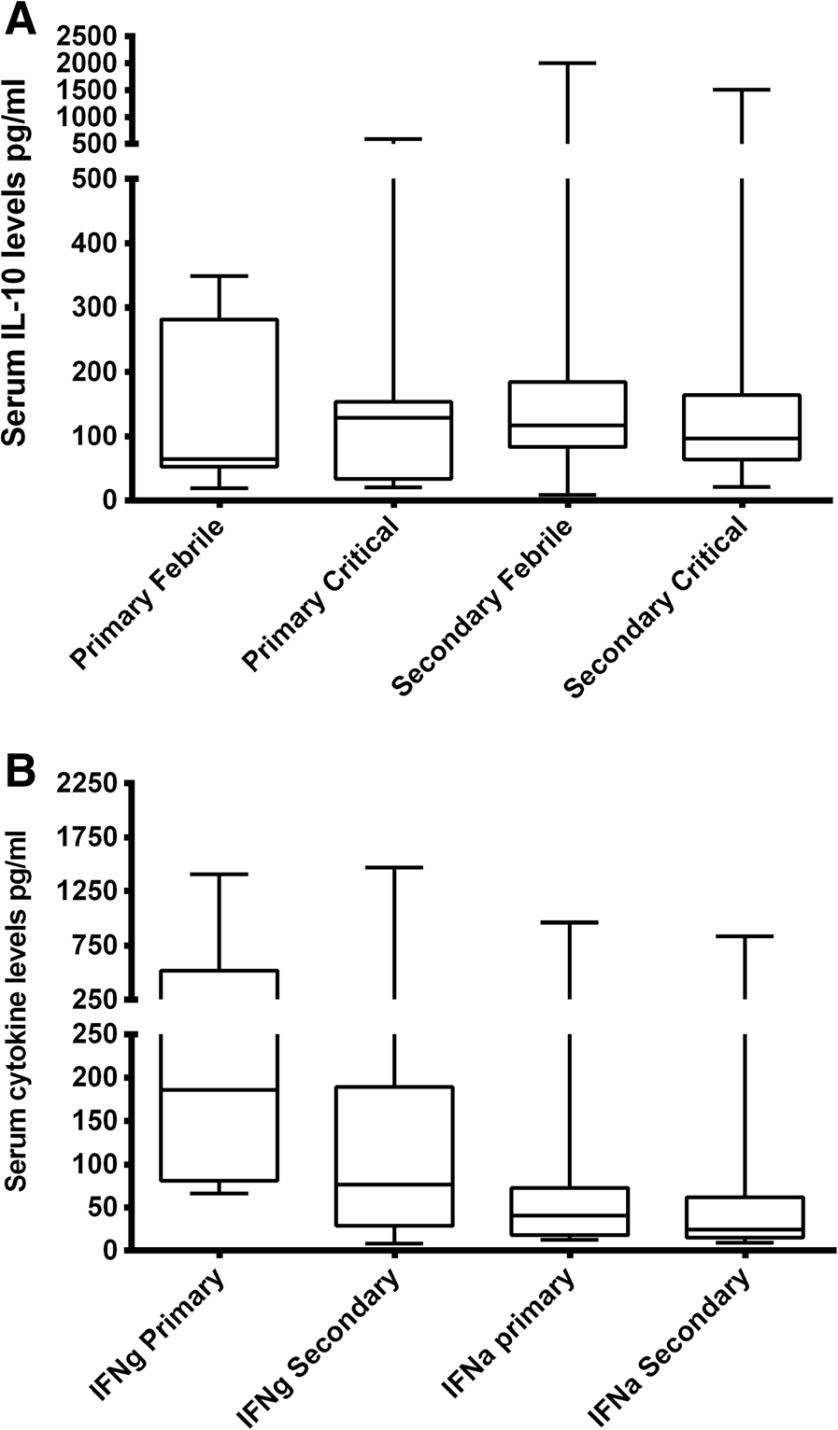
**Serum cytokine levels in primary and secondary dengue infection. A**: Serum IL-10 levels in patients with primary (n = 9) and secondary dengue (n = 56) in the febrile phase and the critical phase. **B**: Serum IFNγ and serum IFNα levels in primary (n = 15) and secondary dengue (n = 63).

50 of our patients had a primary dengue infection whereas 209 had a secondary dengue infection. IL-10 levels were significantly higher (p = 0.01) in those with primary dengue (mean 363, SD ± 923.6 pg/ml) than those with secondary dengue (mean 161.1, SD ± 298.6 pg/ml). In primary dengue infection serum IL-10 was slightly lower in the febrile phase (mean 135.8, SD ± 129.7 pg/ml) when compared to the critical phase (mean 154. 2, SD ± 183.2 pg/ml) although not statistically significant. On the other hand, serum IL-10 levels slightly decreased in the critical phase in those with secondary dengue infection (168.3, SD ± 243.7 pg.ml) when compared to the febrile phase (mean 198.1, SD ± 293.7 pg/ml) (Figure 3A). There was no difference in the IL-10 levels in patients with primary dengue who had severe dengue (mean 162.1, SD ± 103.5 pg/ml) when compared to those with non severe dengue (mean 194.2, SD ± 151.1 pg/ml). However, serum IL-10 was higher in patients with severe secondary dengue infection (mean 329.1, SD ± 647.1 pg/ml) when compared to those with non severe dengue (mean 164, SD ± 155.1 pg/ml) (data not shown). Serum IFNγ levels were significantly higher (p = 0.007) in patients with primary dengue (mean 361.6, SD ± 399.6) when compared to those with secondary dengue infection (mean 161, SD ± 246.3) (Figure 3B). No such difference was seen in serum IFNα levels (p = 0.31). In contrast, serum MIF levels were significantly higher (p = 0.007) in patients with secondary dengue (mean 115,276, SD ± 538,567) when compared to those with primary dengue (mean 12669, SD ± 8.240).

### Usefulness of serum IL-10 levels in the febrile phase as a potential biomarker

Although serum IL-10 levels were found to be associated with severe clinical disease and also significantly correlated with other parameters of severe clinical disease such as liver transaminases, IL-10 levels did not have a good predictive value in discriminating those who were likely to develop severe disease (AUC = 0.66) (Figure 1C). When using the IL-10 cut-off value as 115.8 pg/ml (mean + 2SD of the serum IL-10 values in healthy controls), the odds ratio of developing severe dengue was 2.77 (95% CI 1.386 to 5.520). At the cut-off value of 115.86 pg/ml the specificity was 89.9%, but the sensitivity was only 23.8% indicating that serum IL-10 values are not suitable to be used as a robust biomarker in this population.

## Discussion

We have attempted to determine if cytokines such as IL-10, IFNγ and MIF, which were suggested as potential biomarkers by others, could be used as biomarkers in a large cohort of patients with acute dengue infection in Sri Lanka [9,11,12]. We obtained the first blood sample from patients on 4 to 5 day of illness as adult individuals with suspected dengue infection rarely seek medical treatment before this. We found that serum IL-10 levels indeed were significantly higher in patients with severe dengue and also significantly associated with other disease severity markers such as liver transaminase levels. Serum IL-10 has also been shown to be associated with severe disease in other acute viral infections such as influenza [16].

Although many studies carried out in relatively small patient groups have suggested that IL-10 could possibly be used as a biomarker to predict severe clinical disease [6,17,18], we found that serum IL-10 alone did not have a good discriminatory value (AUC = 0.66). A recent study in 52 patients with acute dengue infection (which included 3 patients with severe dengue) has shown that serum IL-10 levels along with platelet counts were two of the most important parameters associated with DHF [17]. However, when validating the use of serum IL-10 in a larger population of dengue patients, we found that serum IL-10 is unlikely to be useful as a biomarker because of its variability. For instance, when we used the serum IL-10 cut-off value as 115.8 pg/ml (mean + 2SD of the serum IL-10 values in healthy controls), 15 patients with SD had values less than this value. We feel that the wide range of IL-10 and other cytokines seen in patients with severe dengue and milder forms of dengue is due to the complex nature of this disease. Although the critical phase has shown to be associated with rising of the haematocrit, reduction of platelet counts along with clinical fluid accumulation, the exact time the patient enters the critical phase may be sometimes difficult to determine with these clinical parameters alone. Furthermore, the probability of a patient developing shock would also depend on the fluid management regime and how well the patient is monitored. Therefore, there are many confounding factors when trying to use cytokines to determine progression to severe dengue.

As dengue is a very dynamic disease, we determined serum IL-10 levels and MIF levels in 65 patients during the febrile phase and critical phase, hoping to gain a better insight into how these cytokines change in patients with SD and non SD. Of these 65 patients, serum IL-10 levels rose in the second sample in 17 patients, while in 48 patients the IL-10 levels fell. The serum MIF levels did not follow the same pattern as in some patients who had an increase in serum IL-10 in the second sample, MIF levels fell and vice versa. Furthermore, the patients in whom serum IL-10 levels rose did not have more severe clinical disease than those who had decreasing serum IL-10 levels. However, similar to the observations in the febrile phase, serum IL-10 levels in the critical phase was associated with SD (p = 0.01). However, we believe that obtaining samples on just 2 days in acute dengue infection would be unsuitable to determine possible biomarkers as it is a very dynamic disease. Although we obtained the first sample on day 4–5 of illness, this time point might still be too late in the illness to identify potential biomarkers to predict severe disease. In addition, as investigations such as serial ultra sound imaging of the abdomen could not be done due to limited resources there is a possibility that the severity was not identified correctly in all patients. A recent study by Rathnakrishnan et al., which investigated cytokine and chemokine patterns in three phases of dengue showed that marked differences in cytokine levels were seen based on the phase of dengue infection [12]. They found that MIP-1β and IP-10 levels were overall higher in the febrile and critical phases of dengue and serum IL-10 levels remained unchanged in patients with dengue with warning signs, whereas levels decreased in those without warning signs [12]. Therefore, their data too appears to support that IL-10 appears to have a role in the pathogenesis of dengue.

However, since in our previous studies [13] and several others have shown that IL-10 is associated with severe clinical disease, it would be crucial to determine the role of IL-10 in the pathogenesis of dengue infections.

We found that serum IL-10 levels significantly correlated with both ALT and AST levels and the association was stronger with serum ALT levels. AST and ALT are both considered as liver transaminases and they are released from hepatocytes. ALT is considered as a more specific marker of liver inflammation than AST, as AST is also released from other sites including skeletal muscles, heart, kidneys and brain. Therefore, since the correlation of serum IL-10 levels was more with ALT than with AST, it appears that it is more likely to be associated with liver inflammation. Therefore, association of serum IL-10 with ALT may signify liver injury and the rise in IL-10 could be possibly secondary to liver inflammation and be protective.

## Conclusions

In summary, we have determined whether serum IL-10 and several other cytokines could be used as potential biomarkers to predict severe dengue infection, in a large cohort of patients with a varying severity of acute dengue infection. We found that serum IL-10 and IFNγ were significantly elevated in patients with severe dengue. Serum IL-10 levels positively and significantly correlated with liver transaminases and inversely correlated with white cell counts and lymphocyte counts. However, serum IL-10 had a poor discriminatory value in identifying those with severe dengue and non severe dengue and therefore, is unlikely to be useful as a robust biomarker in this population.

## Competing interests

The authors declare that they have no competing interest.

## Authors’ contributions

GNM and GSO were involved in study design, analysis of data and writing the manuscript. LG, SF, LA, MS and KN collected data and carried out the experiments. TC and SDJ were involved in study design and analysis of data. All authors read and approved the final manuscript.

## Pre-publication history

The pre-publication history for this paper can be accessed here:

http://www.biomedcentral.com/1471-2334/13/341/prepub
